# Inverse association between age and risk of lymph node metastasis in patients with early gastric cancer: a surveillance, epidemiology, and end results analysis

**DOI:** 10.7150/jca.94542

**Published:** 2024-03-25

**Authors:** Suya Pang, Weijun Wang, Jianning Zhou, Xin Jiang, Rong Lin

**Affiliations:** Department of Gastroenterology, Union Hospital, Tongji Medical College, Huazhong University of Science and Technology, Wuhan 430022, China.

**Keywords:** early gastric cancer, age, lymph node metastasis, postoperative survival

## Abstract

**Background:** Recent studies have shown that young patients with gastric cancer are at a more advanced stage and have poor survival, but the cause is still unclear. The prognosis of gastric cancer is closely related to LNM, but the relationship between age and LNM in early gastric cancer (EGC) is currently unclear. Therefore, we aimed to study the relationship between age and the risk of LNM in EGC.

**Materials and Methods:** We screened out patients with EGC who underwent surgery from the SEER research database from 1975 to 2016, and retrospectively analyzed the proportion of LNM in different age groups. We grouped age into 18-39, 40-49, 50-59, 60-69, 70-79, and ≥ 80 years old, and used univariate analysis and multivariate logistic regression to analyze the correlation between age and LNM.

**Results:** We included 9231 patients with EGC, with LNM rates of 20.3%, 23.3%, 21.0%, 19.8%, 18.1%, and 13.2% in the age groups of 18-39 years old (2.3%), 40-49 (6.1%), 50-59 years old (15.7%), 60-69 years old (24.8%), 70-79 years old (27.2%) and ≥80 years old (23.9%), respectively. We found that when older than 39 years old, the risk of LNM and postoperative survival time of EGC patients decrease (*p*<0.001). Multivariate analysis results showed that age, tumor size, the number of retrieved lymph nodes (rN), tumor grade, and tumor location were related to LNM.

**Conclusions:** This study found that age in patients with EGC was inversely related to the risk of LNM, and positively correlated with postoperative survival. For older patients with EGC, endoscopic treatment is more appropriate. For young patients with EGC, LNM should be considered when choosing endoscopic treatment.

## Introduction

Gastric cancer (GC) is the fifth most common cancer (fourth for male and seventh for female) and the third most fatal cancer (third for male and fifth for female) in the world.^1^ The incidence of GC is increasing year by year and increases with age.^2^-^4^ Although the increase in the incidence of GC is not obvious in young people, compared with middle-aged and elderly patients, young patients have worse survival and are more likely to be in more advanced stages (18-25 *vs* 26-40)^5^.

Compared with the older population, the growth of GC patients in the younger population is not obvious, the reason may be that young people are more likely to suffer from GC in an earlier stage or precancerous state, which is hard to detect. Another reason may be that young people are not included in the routine endoscopic screening population, so compared with the elderly, young people are less likely to undergo endoscopy. However, the reasons for the poor survival of young patients need to be further studied.

Lymph node metastasis (LNM) of GC is related to prognosis, and this view has become a consensus. 6 However, the relationship between age and lymph node metastasis of GC is still controversial. Some reports indicate that young patients with GC have a higher risk of metastasis 7, 8, but other studies have reported that there is no significant difference in the incidence of LNM between young patients and elderly patients 9, 10. Therefore, we determine to clarify the LNM in different age groups, which is closely related to the choice of subsequent treatment.

Regarding the treatment of GC, it is completely different for the treatment strategies of early gastric cancer (EGC) and advanced gastric cancer 11. Different treatment is mainly selected by the LNM rate. Compared with advanced gastric cancer, the LNM rate of EGC is lower 12. Therefore, EGC is widely treated by endoscopic resection and surgical treatment is often selected for treating advanced gastric cancer 13. Compared with surgical treatment, endoscopic treatment has the advantages of reducing the trauma to the patient, the length of hospitalization, fewer complications, better quality of life and lower costs 14, but it cannot clear the lymph nodes. Therefore, studying the risk factors of LNM in EGC patients can better determine the applicability of endoscopic resection. However, the current view on the relationship between age and LNM in EGC is not uniform 15-17.

This study aims to discover the relationship between age and the risk of LNM and prognosis in EGC through a large population-based study, so as to determine the efficacy and safety of endoscopic resection in patients with EGC.

## Materials and Methods

### Patients

In this study, we included patients with EGC from the SEER database. No informed consent from patients or institutional review board approval was required for this study. In the present study, EGC was defined as EGC confined to the mucosa or submucosa regardless of LNM status. The inclusion criteria used in this study were as follows: (1) patients diagnosed with differentiated GC between 1975 and 2016, aged 18 or older; (2) GC was the first diagnosed primary tumor; (3) surgery was performed for histologically confirmed GC; (4) The staging of was Tis or T1, that is EGC; (5) the number of retrieved lymph nodes (rN) were available; and (6) The number of LNM was determined. We excluded cases of uncertain lymphatic metastasis and non-early gastric cancer cases. And all patients were randomly divided into two groups, namely dataset 1 and dataset 2, to verify the conclusion.

### Variables and outcomes

In the study, the race of the patients was recorded as white, black, other (mainly including American Indian, Asian, and Pacific Islander) or unknown. Sex was recorded as female or male. Location of primary tumor was classified into three different sites: cardia, fundus, body, gastric antrum, pylorus, lesser curvature, greater curvature, overlapping lesion or unknown. The EGC tumor sizes were classified into four groups: ≤1 cm, ≤2 cm, ≤3 cm, and >3 cm. The invasion depth of EGC was coded as Tis stage and T1 stage. The number of rN of EGC were divided into two groups: 1-11 and no less than 12. Age was classified as a categorical variable, namely 18-39 years old, 40-49 years old, 50-59 years old, 60-69 years old, 70-79 years old and no less than 80 years old. The primary outcomes were LNM rate and risk of LNM, and the secondary outcomes were overall survival (OS) and cancer-specific survival (CSS). OS was defined as death regardless of causes and CSS was defined as death due to EGC. Patients who were still alive were censored at the date of last contact.

### Statistical analysis

All analyses were stratified by age. Categorical variables are expressed as numbers with percentages, and chi-square test or Fisher's exact test is used. Logistic regression analysis was used to test the correlation between potential risk factors and LNM. The multivariate logistic regression analysis of age and LNM included age, race, gender, year of diagnosis, tumor location, tumor size, depth of invasion, and rN number. First, age is used as a categorical variable. Compared with patients aged 18-39, calculate the OR of each age category and its 95% confidence interval (CI). Next, by using age as an ordinal categorical variable in a multivariate logistic model, the p-value of the association between the increase in diagnostic age and the risk of LNM is estimated. Survival curves were compared using the Kaplan-Meier method and log-rank test. P<0.05 was considered statistically significant. All statistical analysis was performed using SPSS version 24 (SAS Institute Inc, Cary, NC).

## Results

### Clinical characteristics of patients with EGC

A total of 9231 EGC cases treated by surgery were included in the study. The clinical and pathological characteristics stratified by age were showed in Table 1. The patients in age groups 18-39 years, 40-49 years, 50-59 years, 60-69 years, 70-79 years and ≥80 years were 212 cases (2.3%), 567 cases (6.1%), 1445 cases (15.7%), 2293 cases (24.8%), 2507 cases (27.2%), and 2207 cases (23.9%), respectively. Of 9231 patients, 2214 (22.7%) had positive lymph nodes, and the proportion of LNM was 20.3% in 18-39 years, 23.3% in 40-49 years, 21.0% in 50-59 years, 19.8% in 60-69 years, 18.1% in 70-79 years, and 13.2 % in ≥80 years. There were more male patients than female patients (59.3% vs 40.7%). The patients belong to T1 stage accounts for 97.9% and the Tis stage 2.1%.

### The age of EGC patients older than 39 years was inversely proportional to the risk of LNM

**Figure 1:**
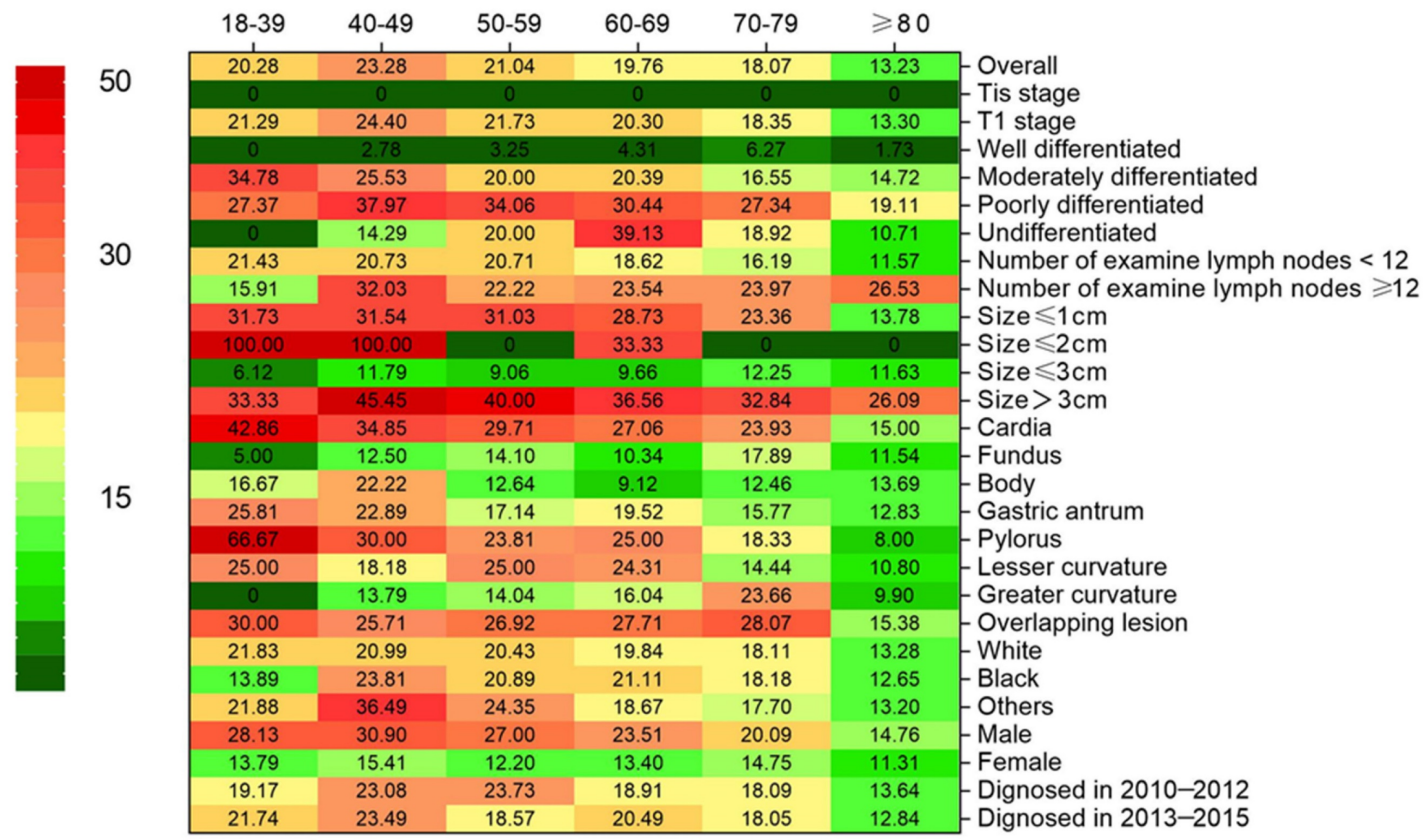
Heatmap showing rate of lymph node metastasis (LNM) of early gastric cancer among patients aged 18-39, 40-49, 50-59, 60-69, 70-79 and ≥80 years stratified by different characteristics, respectively.

We firstly compared LNM rate among patients in different age groups to evaluate the association between age and LNM. There was an inverse correlation between age over 39 years and risk of LNM (**Table 1, and Figure 1**). The rate of LNM was highest (23.3%) in patients aged 40-49 years, and lowest (13.2%) in ≥80 years age group.

**Figure 2:**
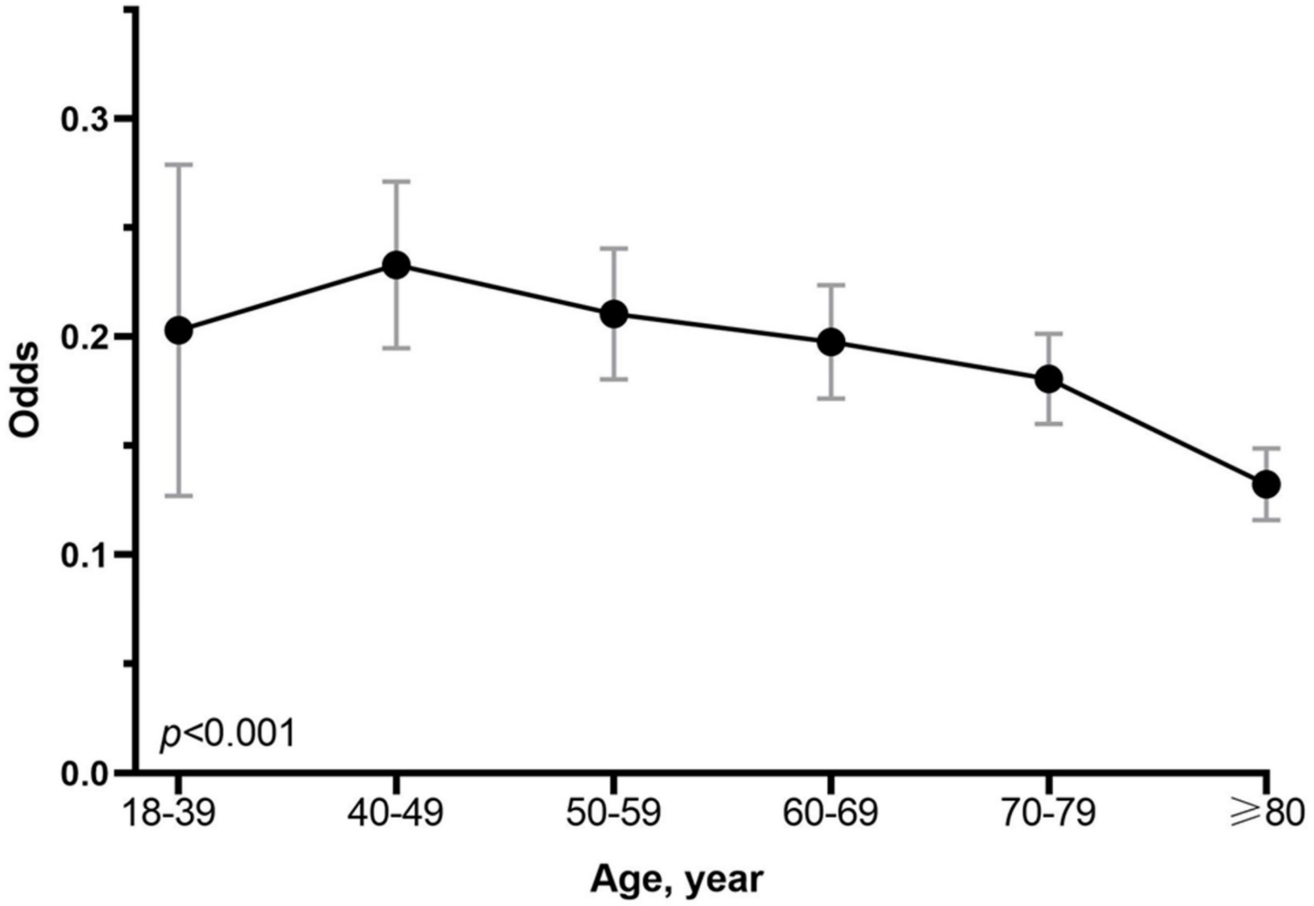
Association between odds of LNM and age at diagnosis in patients with early gastric cancer. The p value for linear trend of the log odds of lymph node metastasis against the numerical code used for age categories was tested using score statistics and its variance.

The score test for trend shown in Figure 2 indications that the increase in age was significantly correlated with the decrease in the probability of LNM (*p* < 0.001). The analysis of dataset 1 and dataset 2 yielded the same results (**Supplementary Table 1, 2 and Supplementary Figure 1**). In addition, we conducted a subgroup analysis to test whether similar trends can be observed in groups stratified by T stage, grade, number of rN, size, location, race, and sex. Table 2 shows the rate of LNM stratified by the T stage. There was no patient in Tis stage who had lymph node metastasis. Compared with other groups, the incidence of LNM was the highest among T1 patients aged 40-49 years, with the trend being significant (p < 0.001). For other subgroup analysis, patients ≥ 80 years old have the lowest and patients aged 40-49 years or 18-39 years had the highest incidence of LNM in almost all subgroups analyzed, except for the well-differentiated grade, the tumor size between 2cm and 3cm, GC in gastric fundus or body (**Figure 1**).

**Figure 3:**
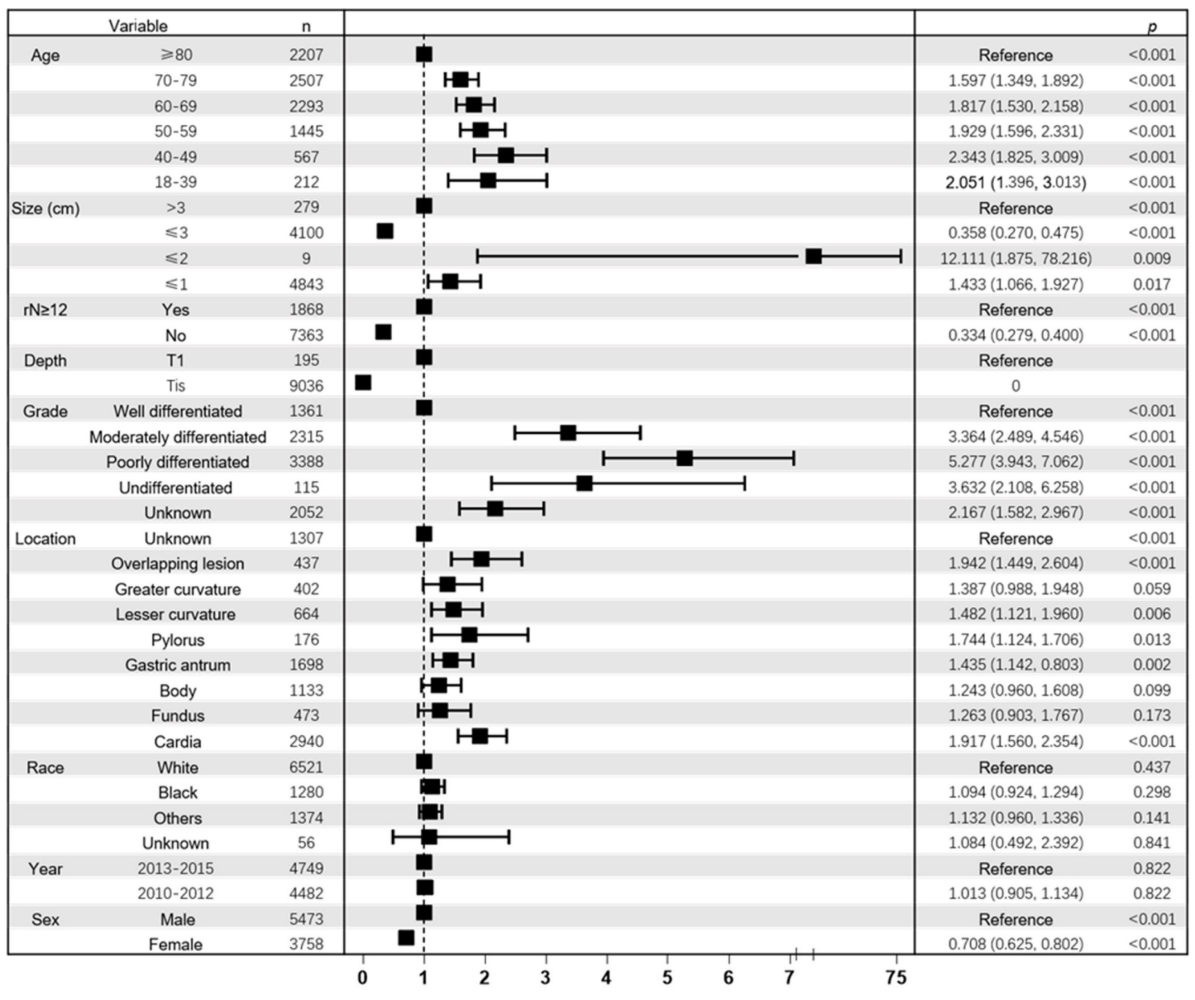
Forest plot showing results of multivariate logistic regression model for identifying potential risk factors for LNM in EGC patients. Abbreviation: rN, number of retrieved lymph nodes

In addition, multivariate analysis showed a significant correlation between age and LNM. The covariates in the adjusted model include the T stage, grade, number of rN, size, location, race and sex. As shown in the Figure 3, compared with patients aged ≥80, patients aged 18-39 years (OR,2.051; 95% CI, 1.396-3.013; p < 0.001), 40-49 years (OR, 2.343; 95% CI, 1.825-3.009; p < 0.001), 50-59 years (OR, 1.929; 95% CI, 1.596-2.331; p < 0.001), 60-69 years (OR, 1.827;95% CI, 1.530-2.158; p < 0.001), and 70-79 years (OR, 1.597; 95% CI, 1.349-1.892; p < 0.001) had higher risk of LNM. And inverse correlation (p < 0.001) between age and LNM could be seen when the variable age was included as an ordered categorical variable in the multivariate logistic model. And the multivariate analysis of datasets 1 and 2 also confirmed the inverse correlation between age and risk of LNM (**Supplementary Figure 2**).

### Postoperative survival rate of EGC according to age

**Figure 4:**
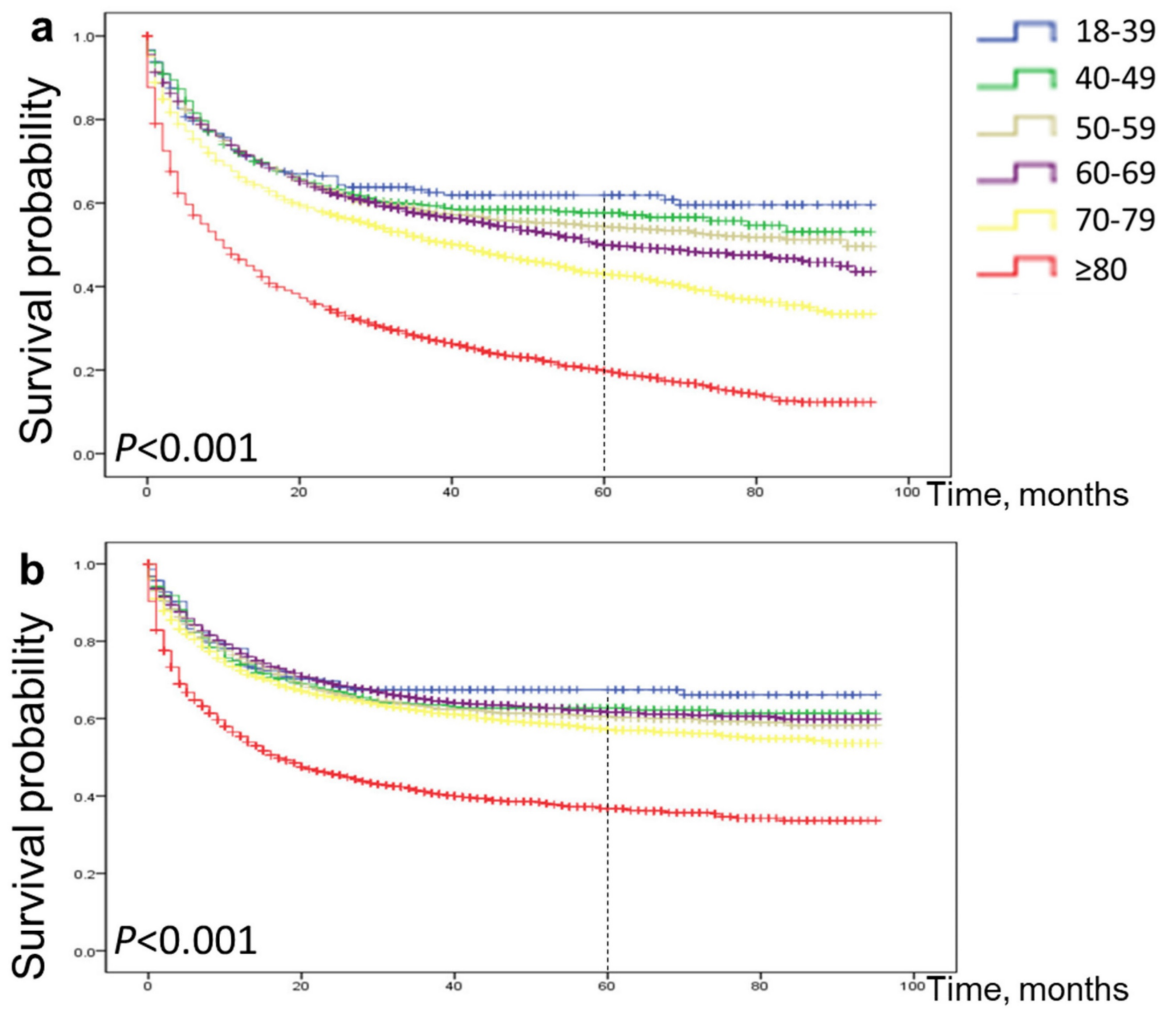
Comparison of cause-specific survival and overall survival among patients with early gastric cancer aged 18-39, 40-49, 50-59, 60-69, 70-79 and ≥80 years. Overall survival (a) and cause-specific survival (b).

As shown in the Figure 4a, 5-year OS for patients aged 18-39 years, 40-49 years, 50-59 years, 60-69 years, 70-79 years and ≥80 years were 62%, 57%, 54%, 50%, 43% and 20%, respectively. Similarly, as shown in Figure 4b, 5-year CSS for patients aged 18-39, 40-49, 50-59, 60-69, 70-79 and ≥80 years were 67%, 63%, 60%, 62%, 57% and 37%, respectively. (all p < 0.001). The probability of survival after surgery decreases with age.

## Discussion

The study showed that the age of patients with EGC was inversely related to the risk of LNM when the age was older than 39 years old, and this was also true for different genders, races, different lymph node examination numbers, and T1 stage, located in cardia, size ≤1cm and >3cm, moderately differentiated and poorly differentiated groups. With respect to the prognosis, age was proportional to the postoperative mortality of patients with EGC. The OS and CSS were the worst for patients not less than 80 years old.

Compared with older patients, patients in the 40-49 age group had the highest proportion of LNM. Previous studies have found that the LNM rate of EGC ranges from 10% to 42% 18-21, which is consistent with the results of this study (22.7%). In current articles on the risk of LNM in EGC, most of the age is divided into only two age groups, and the relationship between age and lymph node metastasis is controversial. The study of Wu et al. showed that compared with people not older than 60 years, people older than 60 have lower lymph node metastasis 22, while some other studies have the opposite results 23, 24. Based on a large sample size, our study comprehensively analyzed the relationship between age and the risk of LNM in EGC from various aspects.

Besides, multivariate analysis found that tumor size, grade, overlapping lesion or located in cardia, male, and rN ≥12 were also risk factors for LNM in EGC. And previous studies have also confirmed that tumor size, depth of invasion, degree of differentiation, and vascular invasion are related to LNM of EGC 19, 21, 22.

In addition, we found that the older the age, the worse the prognosis after surgery. Although patients in the 40-49 age group had the highest risk of LNM, patients ≥80 years of age have the shortest postoperative survival cycle, in the comparison of cause-specific survival and overall survival. Jeung Hui Pyo et al. also found that age is a risk factor affecting the postoperative survival of EGC.^13^ Although the risk of LNM is related to the prognosis of surgical patients, the surgical prognosis of elderly patients is affected by many other factors, such as the comorbidity index, the performance index, tumor morphology. 13 Therefore, for elderly patients with EGC, surgery may not be the best treatment.

The current research has certain limitations. First, lymphatic vessel involvement (LVI) is a high-risk factor for LNM, which was not evaluated in this study. For EGC patients diagnosed at different ages, whether there are differences in LVI, further research is needed. Secondly, due to the low sample size of patients aged 18-29 years, patients aged 18-29 years old and patients aged 30-39 years old were combined into one group. However, LNM accounts for 24.5% of patients aged 18-29 years, which is still higher than that of patients ≥40 years old. Third, this study is based on a retrospective cohort study from the registry database. Therefore, our findings should be interpreted with caution and validated in another prospective patient cohort.

So far, this study is a comprehensive study involving 9231 EGC patients, aiming to study the relationship between the age of onset of EGC and the risk of LNM. The study demonstrated a negative correlation between age at diagnosis and LNM, even if other risk factors were adjusted in multivariate logistic regression. And we found that age is inversely proportional to postoperative survival period. These findings suggest that the age of diagnosis should be taken into consideration when assessing the risk of LNM in EGC patients before choosing endoscopic resection and surgery. And for elderly patients with EGC, we suggest that endoscopic treatment is a better treatment.

## Conclusions

This study suggests that, age is inversely proportional to the LNM of EGC when older than 39 years old, and directly proportional to the postoperative survival of EGC. Endoscopic treatment may be more suitable for older patients.

## Supplementary Material

Supplementary figures and tables.

## Figures and Tables

**Table 1:** Baseline characters of early gastric cancers by age at diagnosis.

		Age						*p*
	Total	18-39	40-49	50-59	60-69	70-79	80+	
Total	9231	212 (2.3)	567 (6.1)	1445 (15.7)	2293 (24.8)	2507 (27.2)	2207(23.9)	
Sex								<0.001
Female	3758 (40.7)	116 (54.7)	279 (49.2)	582 (40.3)	851 (37.1)	949 (37.9)	981 (44.4)	
Male	5473 (59.3)	96 (45.3)	288 (50.8)	863 (59.7)	1442 (62.9)	1558 (62.1)	1226 (55.6)	
Race								0.002
White	6521 (70.6)	142 (67.0)	405 (71.4)	1013 (70.1)	1623 (70.8)	1734 (69.2)	1604 (72.7)	
Black	1280 (13.9)	36 (17.0)	84 (14.8)	225 (15.6)	341 (14.9)	341 (13.6)	253 (11.5)	
Others	1374 (14.9)	32 (15.1)	74 (13.1)	193 (13.4)	316 (13.8)	418 (16.7)	341 (15.4)	
Unknown	56 (0.6)	2 (0.9)	4 (0.7)	14 (1.0)	13 (0.6)	14 (0.6)	9 (0.4)	
Year of diagnosis								0.031
2010-2012	4482 (48.6)	120 (56.6)	286 (50.4)	691 (47.8)	1063 (46.4)	1244 (49.6)	1078 (48.8)	
2013-2015	4749 (51.4)	92 (43.4)	281 (49.6)	754 (52.2)	1230 (53.6)	1263 (50.4)	1129 (51.2)	
Location								<0.001
Cardia	2940 (31.8)	35 (16.5)	132 (23.3)	488 (33.8)	850 (37.1)	815 (32.5)	620 (28.1)	
Fundus	473 (5.1)	20 (9.4)	32 (5.6)	78 (5.4)	116 (5.1)	123 (4.9)	104 (4.7)	
Body	1133 (12.3)	36 (17.0)	81 (14.3)	182 (12.6)	274 (11.9)	297 (11.8)	263 (11.9)	
Gastric antrum	1698 (18.4)	31 (14.6)	83 (14.6)	210 (14.5)	374 (16.3)	501 (20.0)	499 (22.6)	
Pylorus	176 (1.9)	3 (1.4)	10 (1.8)	21 (1.5)	32 (1.4)	60 (2.4)	50 (2.3)	
Lesser curvature	664 (7.2)	16 (7.5)	33 (5.8)	108 (7.5)	144 (6.3)	187 (7.5)	176 (8.0)	
Greater curvature	402 (4.4)	16 (7.5)	29 (5.1)	57 (3.9)	106 (4.6)	93 (3.7)	101 (4.6)	
Overlapping lesion	437 (4.7)	10 (4.7)	35 (6.2)	78 (5.4)	83 (3.6)	114 (4.5)	117 (5.3)	
Unknown	1307 (14.2)	44 (20.8)	132 (23.3)	223 (15.4)	314 (13.7)	317 (12.6)	277 (12.6)	
Size, cm								<0.001
≤1	4843 (52.5)	104 (49.1)	279 (49.2)	696 (48.2)	1079 (47.1)	1190 (47.5)	1495 (67.7)	
≤2	9 (0.1)	1 (0.5)	3 (0.5)	0	3 (0.1)	1 (0.0)	1 (0.0)	
≤3	4100 (44.4)	98 (46.2)	263 (46.4)	684 (47.3)	1118 (48.8)	1249 (49.8)	688 (31.2)	
>3	279 (3.0)	9 (4.2)	22 (3.9)	65 (4.5)	93 (4.1)	67 (2.7)	23 (1.0)	
T stage								<0.001
Tis	195 (2.1)	10 (4.7)	26 (4.6)	46 (3.2)	62 (2.7)	39 (1.6)	12 (0.5)	
T1	9036 (97.9)	202 (95.3)	541 (95.4)	1399 (96.8)	2231 (97.3)	2468 (98.4)	2195 (99.5)	
Grade								<0.001
Well differentiated	1361 (14.7)	31 (14.6)	108 (19.0)	246 (17.0)	394 (17.2)	351 (14.0)	231 (10.5)	
Moderately differentiated	2315 (25.1)	23 (10.8)	94 (16.6)	300 (20.8)	559 (24.4)	707 (28.2)	632 (28.6)	
Poorly differentiated	3388 (36.7)	95 (44.8)	237 (41.8)	549 (38.0)	795 (34.7)	885 (35.3)	827 (37.5)	
Undifferentiated	115 (1.2)	0	7 (1.2)	20 (1.4)	23 (1.0)	37 (1.5)	28 (1.3)	
Unknown	2052 (22.2)	63 (29.7)	121 (21.3)	330 (22.8)	522 (22.8)	527 (21.0)	489 (22.2)	
Number of examined lymph nodes								<0.001
<12	7363 (79.8)	168 (79.2)	439 (77.4)	1130 (78.2)	1762 (76.8)	1902 (75.9)	1962 (88.9)	
≥12	1868 (20.2)	44 (20.8)	128 (22.6)	315 (21.8)	531 (23.2)	605 (24.1)	245 (11.1)	
LNM								<0.001
No	7554 (77.3)	169 (79.7)	435 (76.7)	1141 (79.0)	1840 (80.2)	2054 (81.9)	1915 (86.8)	
Yes	2214 (22.7)	43 (20.3)	132 (23.3)	304 (21.0)	453 (19.8)	453 (18.1)	292 (13.2)	
Survival years								<0.001
≤1	3397 (36.8)	70 (33.0)	179 (31.6)	426 (29.5)	664 (29.0)	870 (34.7)	1188 (53.8)	
≤3	2269 (24.6)	42 (19.8)	138 (24.3)	371 (25.7)	620 (27.0)	594 (23.7)	504 (22.8)	
≤5	1811 (19.6)	33 (14.7)	120 (20.3)	318 (20.7)	532 (22.1)	545 (20.8)	309 (13.0)	
>5	1746 (18.9)	67 (31.6)	131 (23.1)	343 (23.7)	484 (21.1)	507 (20.2)	214 (9.7)	
Unknown	8 (0.1)	0	1 (0.2)	0	2 (0.1)	2 (0.1)	3 (0.1)	

**Table 2:** Lymph node positivity with age within T stage groups.

	T stage Tis		T stage T1	
Age group, yr	N	LN-positive	N	LN-positive
18-39	10	0	202	43 (21.3)
40-49	26	0	541	132 (24.4)
50-59	46	0	1399	304 (21.7)
60-69	62	0	2231	453 (20.3)
70-79	39	0	2468	453 (18.4)
80+	12	0	2195	292 (13.32)
*P*			<0.001	

Abbreviation: LN, lymph node.
