# Supplementary material for: Inverse association between age and risk of lymph node metastasis in patients with early gastric cancer: a surveillance, epidemiology, and end results analysis
Source: J Cancer. 2024 Mar 25;15(9):2829–36. doi: 10.7150/jca.94542 (PMC10988299; doi:10.7150/jca.94542)
Supplement: Supplementary file 1 — Supplementary figures and tables. [file jcav15p2829s1.pdf]

Supplementary table 1. Baseline characters of early gastric cancers by age at diagnosis of dataset 1.

	Age							*p*
	Total	18-39	40-49	50-59	60-69	70-79	80+	
**Total**	4613	102 (2.2)	311 (6.7)	717 (15.5)	1113 (24.1)	1278 (27.7)	1092(23.7)	
**LNM**								<0.001
**No**	3776 (82.2)	80 (78.4)	236 (75.9)	572 (79.8)	896 (80.5)	1060 (82.9)	952 (87.2)	
**Yes**	817 (17.8)	22 (21.6)	75 (24.1)	145 (20.2)	217 (19.5)	218(17.1)	140 (12.8)	

Supplementary table 2. Baseline characters of early gastric cancers by age at diagnosis of dataset 2.

	Age							*p*
	Total	18-39	40-49	50-59	60-69	70-79	80+	
**Total**	4618	110 (2.4)	256 (5.5)	728 (15.8)	1180 (25.6)	1229 (26.6)	1115(24.1)	
**LNM**								<0.001
**No**	3758 (81.4)	89 (80.9)	199 (77.7)	569 (78.2)	944 (80.0)	994 (80.9)	963 (86.4)	
**Yes**	860 (18.6)	21 (19.1)	57 (22.3)	159 (21.8)	236 (20.0)	235 (19.1)	152 (13.6)	

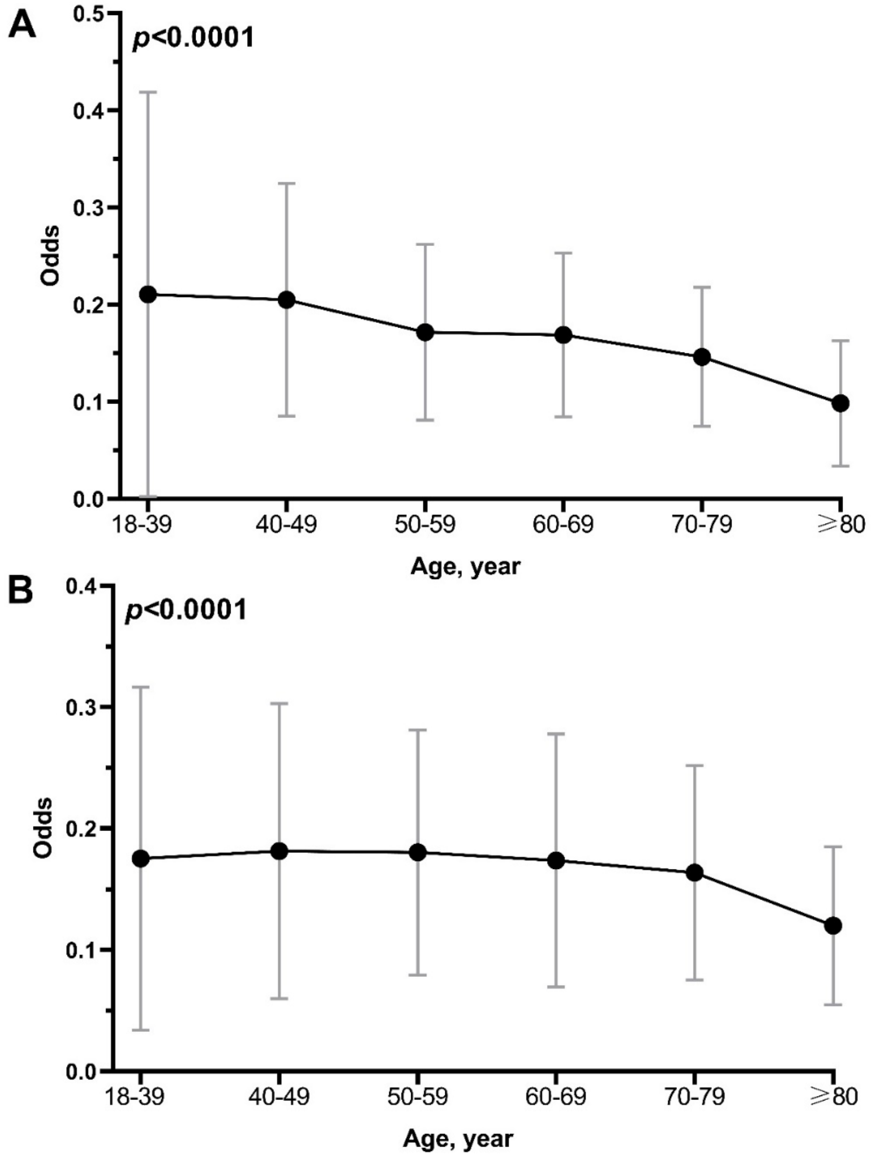

Supplementary figure1, Association between odds of LNM and age at diagnosis in patients with early gastric cancer. The p value for linear trend of the log odds of lymph node metastasis against the numerical code used for age categories was tested using score statistics and its variance. (A, Dataset 1 and B, Dataset 2.)

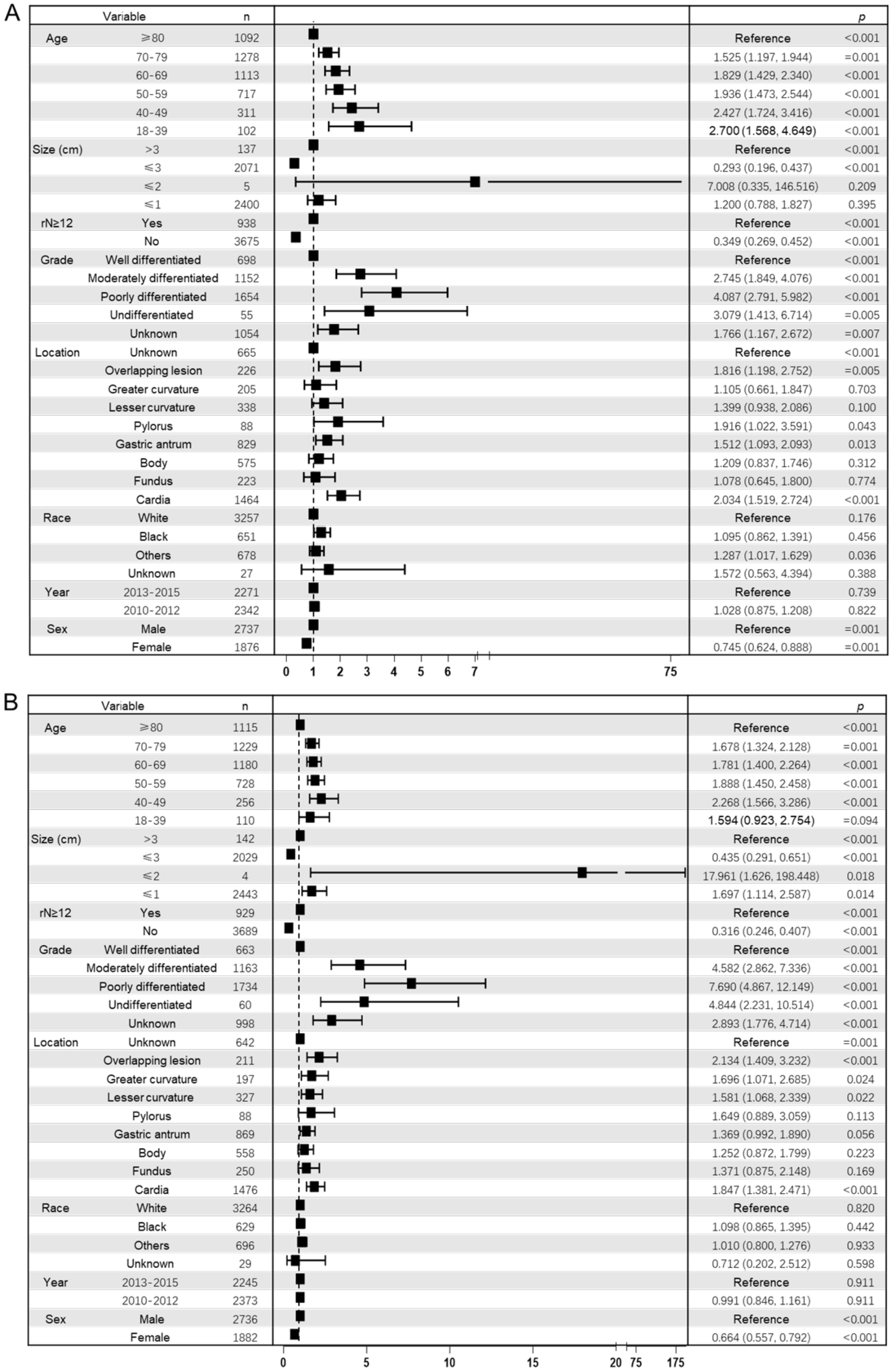

Supplementary figure2, Forest plot showing results of multivariate logistic regression model for identifying potential risk factors for LNM in EGC patients. (A, Dataset 1 and

B, Dataset 2.)

Abbreviation: rN, number of retrieved lymph nodes
